# Molecular Subtypes Are Associated With Clinical Benefit in Cisplatin-Treated Metastatic Urothelial Cancer Patients

**DOI:** 10.1200/PO.24.00209

**Published:** 2024-10-02

**Authors:** Karin Holmsten, Gottfrid Sjödahl, Johan Abrahamsson, Carina Bernardo, Pontus Eriksson, Mattias Höglund, Fredrik Liedberg, Anders Ullén

**Affiliations:** ^1^ Department of Oncology-Pathology, Karolinska Institute, Stockholm, Sweden; ^2^ Department of Oncology, Capio S:t Görans Hospital, Stockholm, Sweden; ^3^ Department of Translational Medicine, Lund University, Malmö, Sweden; ^4^ Department of Urology, Skåne University Hospital, Malmö, Sweden; ^5^ Division of Oncology, Department of Clinical Sciences, Lund University, Lund, Sweden; ^6^ Department of Pelvic Cancer, Genitourinary Oncology and Urology Unit, Karolinska University Hospital, Stockholm, Sweden

## Abstract

**PURPOSE:**

Cisplatin-based combination chemotherapy (CHT) is standard of care in metastatic urothelial cancer (mUC); however, no predictive molecular biomarkers are available for clinical use. The aim of this study was to investigate the impact of molecular subtypes in relation to treatment response and survival in patients with mUC treated with first-line CHT.

**PATIENTS AND METHODS:**

Molecular subtype classification according to the Lund Taxonomy (LundTax) was performed by tumor transcriptomic profiling and immunostaining in a retrospective cohort. Molecular subtypes were investigated in relation to the primary end point overall response rate (ORR) and secondary end points progression-free survival (PFS) and overall survival (OS). Differential gene expression and association to treatment response were explored.

**RESULTS:**

Ninety-five patients with mUC were classified into urothelial-like (Uro, 43%), genomically unstable (GU, 26%), basal squamous-like (Ba/Sq, 20%), mesenchymal-like (Mes-like, 8%), and small cell neuroendocrine-like (Sc/NE, 3%) subtypes. Patients with Mes-like tumors had lower ORR (14%) compared with Uro (70%), GU (77%), Ba/Sq (75%), and Sc/NE (67%; odds ratio, 0.06 [95% CI, 0.01 to 0.54], *P* = .012). Furthermore, patients with Mes-like tumors had significantly shorter PFS (hazard ratio [HR], 5.18 [95% CI, 2.28 to 11.76], *P* < .001) and OS (HR, 3.19 [95% CI, 1.45 to 7.03], *P* = .004). Patients with Uro and GU showed the longest survival. In responders, an enrichment of downregulated stromal- and immune-related genes was seen. Downregulation of interferon-induced transmembrane protein 2 was associated with increased ORR and improved OS.

**CONCLUSION:**

This study identifies different CHT responses by LundTax molecular subtypes in patients with mUC, where the Mes-like subtype was associated with lower response rate and shorter survival.

## Teaser

Molecular subtypes correlate with treatment response and survival in metastatic urothelial cancer.

## INTRODUCTION

Cisplatin-based combination chemotherapy (CHT) demonstrate in patients with locally advanced and/or metastatic urothelial cancer (mUC) an overall response rate (ORR) of 44%-72% and an overall survival (OS) of approximately 15 months.^1-4^ There is a considerable heterogeneity in mUC with regard to de novo and acquired tumor resistance to platinum treatment. Some candidate molecular markers have been identified,^5-7^ but there are presently no predictive biomarkers implemented in clinical use.

**CONTEXT**

**Key Objective**
To investigate the impact of molecular subtypes as predictive molecular biomarker in relation to treatment response in patients with metastatic urothelial cancer (mUC) treated with first-line cisplatin-based combination chemotherapy (CHT).
**Knowledge Generated**
In this study, molecular subtype classification according to the Lund Taxonomy (LundTax) was performed by tumor transcriptomic profiling and immunostaining in a retrospective cohort. The molecular LundTax subtypes correlated with differential response and survival in patients with mUC treated with CHT. Patients with the mesenchymal-like subtype had both significantly lower response rate and shorter survival, while the luminal-like subtypes (urothelial-like and genomically unstable) displayed the best response rates and longest survival.
**Relevance**
This study provides insight that molecular subtypes hold promise as a predictive biomarker for developing precision medicine in mUC. Further validation is needed before clinical implementation, as well as to address the present findings in novel combination strategies including immune checkpoint inhibitors and antibody-drug conjugates.

We have previously suggested a molecular taxonomy for bladder cancer on the basis of transcriptomic profiling, The Lund Taxonomy (LundTax).^8-10^ Other classification systems on the basis of gene expression have also been proposed, including a consensus molecular classification for muscle invasive bladder cancer (MIBC),^11^ well conformed with the LundTax. LundTax subtypes have been developed for identifying stable and distinct states of the cancer cells, whereas the MIBC consensus subtypes reflect encapsulate classification according to several published systems and is driven by expression in the cancer cells as well as in any other cell types present in the analyzed tissue sample. In the LundTax, the two luminal-like subtypes, namely urothelial-like (Uro) and genomically unstable (GU), are characterized by a retained urothelial stratification similar to normal urothelium and by a loss of tissue stratification and signs of having undergone genomic instability, respectively. The LundTax also includes three nonluminal subtypes: basal squamous (Ba/Sq) characterized by squamous differentiation, mesenchymal-like (Mes-like) characterized by a mesenchymal-like state suggestive of partial or full epithelial to mesenchymal transition, and small cell/neuroendocrine-like (Sc/NE) characterized by the presence of a neuroendocrine molecular signature. The consensus subtypes include three luminal-like classes, LumP overlapping with LundTax-Uro, often showing papillary histology, LumU overlapping with LundTax-GU, and LumNS comprising a class of less well-defined luminal tumors. In addition, the consensus subtyping includes a class comprising tumor biopsies dominated by stroma (Stroma-rich), a basal squamous class similar to LundTax-Ba/Sq, and a neuroendocrine-like subtype similar to LundTax-NE-like.

We have shown that the luminal-like subtypes are more responsive to neoadjuvant CHT in MIBC.^12^ However, other classification systems not designed to define the actual cancer cell phenotype^13^ show disparate results on response to CHT in MIBC.^6,11,14-18^ In mUC, molecular subtypes and response prediction in patients treated with systemic therapies have been sparsely investigated,^6,19^ and MIBC classifiers are still not validated in metastatic disease.^20^

In this study, we investigated the distribution of the LundTax molecular subtypes in a population-based mUC cohort and association with response and survival in patients treated with first-line CHT with palliative intent.

## PATIENTS AND METHODS

### Patient Cohort and Outcome Measures

Patients treated with first-line CHT for mUC in Stockholm and the Southern Health Care Region in Sweden between 2003 and 2015 were retrospectively identified. In all, 95 patients were included, for which 86 had full transcriptomic data available (Data Supplement, Fig S1). A comprehensive database was established including relevant clinical data. The trial was approved by the Swedish Ethical Review Authority Dnr 2013/264, 2013/453, and 2017/37.

The primary outcome measure was ORR (complete response [CR] and partial response [PR]) in the LundTax subtypes. Disease control rate (DCR) was defined as CR, PR, and stable disease (SD). Response evaluation was based on computed tomography reported in clinical routine. Secondary outcome measures were progression-free survival (PFS) and OS. PFS was defined as the time from start of first-line CHT to progression or death, whichever came first. OS was defined as the time from start of CHT to death from any cause. Furthermore, outcomes were also analyzed in the MIBC consensus molecular subtypes.

### Pathologic Evaluation and Immunostaining

The pathologic specimens closest in time to start of first-line palliative chemotherapy were selected and re-reviewed. Grading was performed according to the 1999 WHO classification system. Two 1.0-mm tissue cores per tumor, placed in areas rich in viable cancer cells, were used to construct tissue microarrays (TMAs) and stained with a panel of subtype-specific antibodies (Data Supplement, Table S1). For each marker, the mean intensity and percentage of expression in the cancer cells was evaluated and multiplied. Values were averaged per case and combined into subtype scores as follows: Uro, *FGFR3+*, *CCND1+*, *RB1+*, *CDKN2A–p16–*; GU, *FGFR3–*, *CCND1–*, *RB1–*, *CDKN2A–p16+*; Ba/Sq, *KRT14+*, *KRT5+*, *GATA3–*, *FOXA1–*; Mes-like, *EPCAM–*, *CDH1–*, *VIM+*, *ZEB2+*; Sc/NE, *EPCAM+*, *TUBB2B+*, *CDH1–*, *GATA3–*. Immunohistochemistry (IHC) classification was performed such that cases above cutoff for any of the nonluminal subtype scores were classified as the highest of those subtypes, and cases below cutoff for all nonluminal subtype scores were classified as Uro or GU depending on the Uro-GU subtype score.^9^ After staining with subtype-specific antibodies, TMA sections were scanned (Axioscan Z.1, Zeiss, Oberkochen, Germany) and evaluated semiquantitatively as digital images. Antibodies were evaluated on the basis of the percentage and/or intensity of staining of the cancer cells, and the mean score of the two tissue cores per tumor was used. The RNA- and IHC-based LundTax classifications along with the MIBC consensus subtypes are provided as metadata with the published data set.

### Transcriptome Analysis

RNA was extracted from formalin-fixed paraffin-embedded tissue blocks. A tumor-rich area was macrodissected from 4 to 10 sections from tissue blocks (10 μm) and used for RNA extraction with the FFPE RNA isolation kit (Roche, Basel, Switzerland). Isolated total RNA was amplified, labeled, and hybridized to Affymetrix Gene ST 1.0 microarrays. Raw and processed data are available via gene expression omnibus with accession number GSE250194. Classification according to LundTax was performed using a single-sample, rule-based, k-top scoring pairs classifier applied to raw intensity values.^21^ The LundTax subtypes' biology is visualized using various gene signatures, briefly the 141-gene immune and stroma signatures are derived from the ESTIMATE tool,^22^ the Squamous signature is an early bladder squamous cell carcinoma versus urothelial carcinoma (UC) microarray signature,^23^ and the *KRT5/14*/*FOXA1*/*GATA3* are the genes proposed to discriminate basal from luminal UC in the early discussion in the MIBC consensus group.^24^ The remaining signatures were defined by quality threshold clustering^8^ or by hierarchical clustering analysis.^9^ Classification with the MIBC consensus classifier was applied to quantile-normalized data. For heatmap visualization, data were adjusted for labeling batch using ComBat,^25^ quantile-normalized, log-transformed, and median-centered. Differential gene expression analyses were performed with Significance Analysis of Microarrays in Multi Experiment Viewer, using *q* < 0.01 as the significance threshold. Testing for differentially expressed genes (DEGs) was performed by comparing cases with CR or PR to those with SD or progressive disease (PD) in the full data set, as well as separately within luminal (Uro [subdivided into UroA, UroB, and UroC in the RNA classification] and GU) and nonluminal (Ba/Sq, Mes-like, and Sc/NE) subtypes. Top 100 upregulated and downregulated genes were tested for enrichment against established signatures for stromal and immune tumor microenvironments.^22^ Gene set enrichment analysis was performed using the gene set enrichment analysis (GSEA) preranked algorithm.^26^ The ranked output of differential expression analysis between responders and nonresponders were analyzed with 1,000 permutations for enrichment of signatures, of sizes 5-500 genes, contained in mSigDB version 7.1 gene sets H.Hallmark, C2 Curated, and C6.Oncogenic.

### Statistical Analyses

Frequencies and response data were analyzed using Pearson χ^2^ test. Continuous data were categorized into nominal data. A significance level of *P* < .05 was applied. Odds ratios (OR) were estimated with 95% CIs, and univariable and multivariable logistic regressions were performed for nominal data. Time-to-event analyses (PFS and OS) were performed using the log-rank (Mantel-Cox) method and displayed by Kaplan-Meier curves. Hazard ratios (HR) were estimated with 95% CIs. In survival analyses, baseline clinical parameters were adjusted for, using univariable and multivariable Cox proportional hazards (CoxPH) regressions. For univariable and multivariate logistic regressions and CoxPH, the molecular subtype with the best outcome in each separate analysis was chosen as reference. Data were analyzed using SPSS statistics software for Windows (version 26) and R version 4.1.2.

## RESULTS

### Baseline Clinical Characteristics and Treatment

Baseline clinical characteristics and treatment patterns are outlined in Table 1. In all, 95 patients with locally advanced urothelial cancer (16%) or mUC (84%) were treated with first-line CHT with palliative intent. All patients were chemotherapy-naïve, that is, had not previously received any systemic neoadjuvant, adjuvant, or palliative treatment. Forty-one percent of the patients later received further treatment beyond first-line CHT; no patients received immune checkpoint inhibitors (ICIs) or antibody-drug conjugates (ADCs). The type of tumor tissue used for molecular analyses was in 91% derived from the primary tumor (Table 1). Median follow-up time was 12.4 months (range, 0.3-173.8).

**TABLE 1.** Baseline Clinical Characteristics and Treatment Patterns

Characteristic	N = 95
Age (years), median (range)	65.2 (37.7-80.4)
Sex, No. (%)	
Male	65 (68)
Clinical stage, No. (%)	
Locally advanced cT4bN0M0	2 (2)
cN+ (local lymph nodes)	13 (14)
cM+ (nonregional lymph nodes or metastases)	80 (84)
Histology, No. (%)	
Pure urothelial	79 (83)
Mixed histology	12 (13)
Other^a^	4 (4)
Metastatic site, No. (%)	
Locoregional recurrence	20 (21)
Lymph nodes	58 (61)
Lung	34 (36)
Liver	19 (20)
Bone	29 (31)
Other^b^	4 (4)
Visceral metastases,^c^ No. (%)	
No	29 (31)
Yes	66 (69)
ECOG performance status, No. (%)	
0	69 (73)
1	20 (21)
2	5 (5)
Missing	1 (1)
Hb (g/L),^d^ median (range)	12.5 (9.3-15.9)
Primary metastatic disease, No. (%)	
Yes	53 (56)
Primary curative treatments, No. (%)	
Yes	42 (44)
Cystectomy	34 (36)
Nephroureterectomy	6 (6)
Segmental ureterectomy	1 (1)
Radiotherapy	1 (1)
Previous perioperative chemotherapy	0
Type of tissue for molecular analyses,^e^ No. (%)	
TUR-B	54 (57)
Radical cystectomy	27 (28)
Nephroureterectomy	6 (6)
Metastases	4 (4)
Other^f^	4 (4)
First-line chemotherapy regimen, No. (%)	
MVAC	38 (40)
Gemcitabine/cisplatin	50 (53)
Gemcitabine/carboplatin	4 (4)
Other^g^	3 (3)
Cycles, median, No. (range)	5 (1-9)
Reason to stop chemotherapy, No. (%)	
Progressive disease	22 (23)
Adverse events	17 (18)
Patient's wish	4 (4)
Planned stop	50 (53)
Other	2 (2)
Second-line chemotherapy, No. (%)	39 (41)
Third-line chemotherapy,^h^ No. (%)	16 (17)

Abbreviations: ECOG, Eastern Cooperative Oncology Group; 5-FU, 5-fluorouracil; Hb, hemoglobin; ITG, ifosfamide and docetaxel; MVAC, methotrexate, vinorelbine, doxorubicin, and cisplatin; TUR-B, transurethral resection of the bladder.

^a^ Pure squamous cell carcinoma (n = 1), anaplastic carcinoma (n = 1), cancer in situ (n = 1), and missing data (n = 1).

^b^ Peritoneal carcinomatosis (n = 2), skin metastases (n = 1), and pleural fluid (n = 1).

^c^ Nonvisceral metastases include patients with lymph node metastases and/or local recurrence only. Visceral metastases include lung, liver, bone, and other metastases.

^d^ Missing data in one patient, n = 94.

^e^ For all patients except two, tumor tissue sample was taken before start of chemotherapy.

^f^ Local recurrence (n = 2), urethra biopsy (n = 1), and lymph nodes at cystectomy (n = 1).

^g^ MVAC + ITG, n = 2, and cisplatin/5-FU, n = 1.

^h^ Three patients received a fourth line of chemotherapy and one patient received immunotherapy with pembrolizumab as fifth-line systemic treatment.

### Molecular Classification

**FIG 1.**
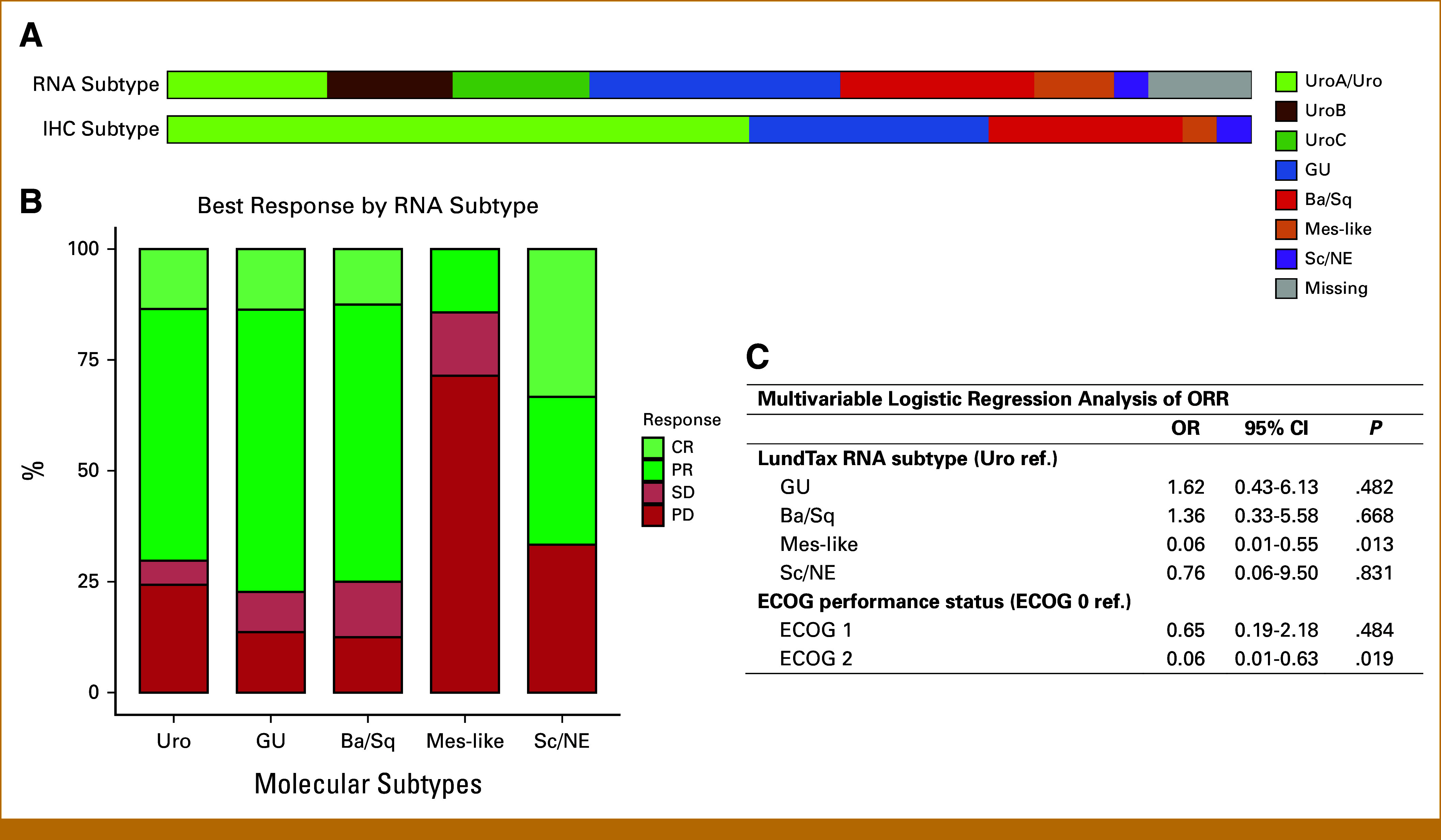
RNA-based molecular subtypes are associated with pathologic response to palliative chemotherapy. (A) Number of patients displaying the different subtypes on the basis of LundTax RNA and IHC subtypes. (B) Stacked barplot displays the proportion achieving CR, PR, SD, or PD by the LundTax RNA subtypes. (C) Multivariable logistic regression showing lower ORR for Mes-like subtype independently of ECOG performance status. For response evaluation n = 85, nine patients had missing extracted RNA and one patient had missing clinical response data. Ba/Sq, basal squamous-like; CR, complete response; ECOG, Eastern Cooperative Oncology Group; GU, genomically unstable; IHC, immunohistochemistry; LundTax, Lund taxonomy; Mes-like, mesenchymal-like; OR, odds ratio; ORR, overall response rate; PD, progressive disease; PR, partial response; Sc/NE, small cell neuroendocrine-like; SD, stable disease; Uro, urothelial-like.

Outcomes were explored according to RNA- (n *=* 86) and IHC-based (N *=* 95) LundTax molecular subtype classifications (Uro [subdivided into UroA, UroB, and UroC by the RNA-based classification], GU, Ba/Sq, Mes-like, and Sc/NE) as illustrated in Figure 1A. The tumors were further analyzed according to the consensus classification system (n = 86).^11^ The concordance between the LundTax RNA- and IHC-based subtypes and the consensus classification are shown in the Data Supplement (Figs S2A and S2B). LundTax molecular subtypes are suggested to be associated with different metastatic sites.^27^ We found that patients with Mes-like tumors had more local recurrences and less visceral metastases compared with the other subtypes (Data Supplement, Table S2).

### Molecular Subtypes and Response

ORR was 70% in the complete cohort. Patients with RNA-based Mes-like subtype had significantly lower ORR, with one of seven (14%) responding, compared with 26/37 (70%) in Uro, 17/22 (77%) in GU, 12/16 (75%) in Ba/Sq, and 2/3 (67%) in Sc/NE (Mes-like *v* non–Mes-like, OR, 0.06 [95% CI, 0.01 to 0.54], *P* = .012; Fig 1B). When the Uro subtype was further subdivided, patients with UroB tumors showed a lower response rate, with ORR in five of 11 patients (46%) compared with 11/14 (79%) in UroA tumors and 10/12 (83%) in UroC tumors (Data Supplement, Table S3). Multivariable logistic regression analysis, including molecular subtypes and clinical baseline characteristics, showed that Eastern Cooperative Oncology Group performance status (ECOG PS) > 1 and Mes-like subtype were independently associated with lower ORR (Fig 1C and Data Supplement, Table S4). For the IHC-based assessment of LundTax, there were no significant differences in ORR. For the consensus subtypes, Ba/Sq showed significant lower ORR compared with the other subtypes, with LumU as reference (Data Supplement, Table S4), and Ba/Sq tumors were also independently associated with lower ORR in multivariable logistic regression analysis including consensus molecular subtypes and ECOG PS (Data Supplement, Table S5). DCR was significantly lower for the Mes-like tumors compared with the other subtypes using the LundTax RNA classification and significantly lower for Ba/Sq using the consensus classification (Data Supplement, Tables S3 and S6).

### Molecular Subtypes and Survival

**FIG 2.**
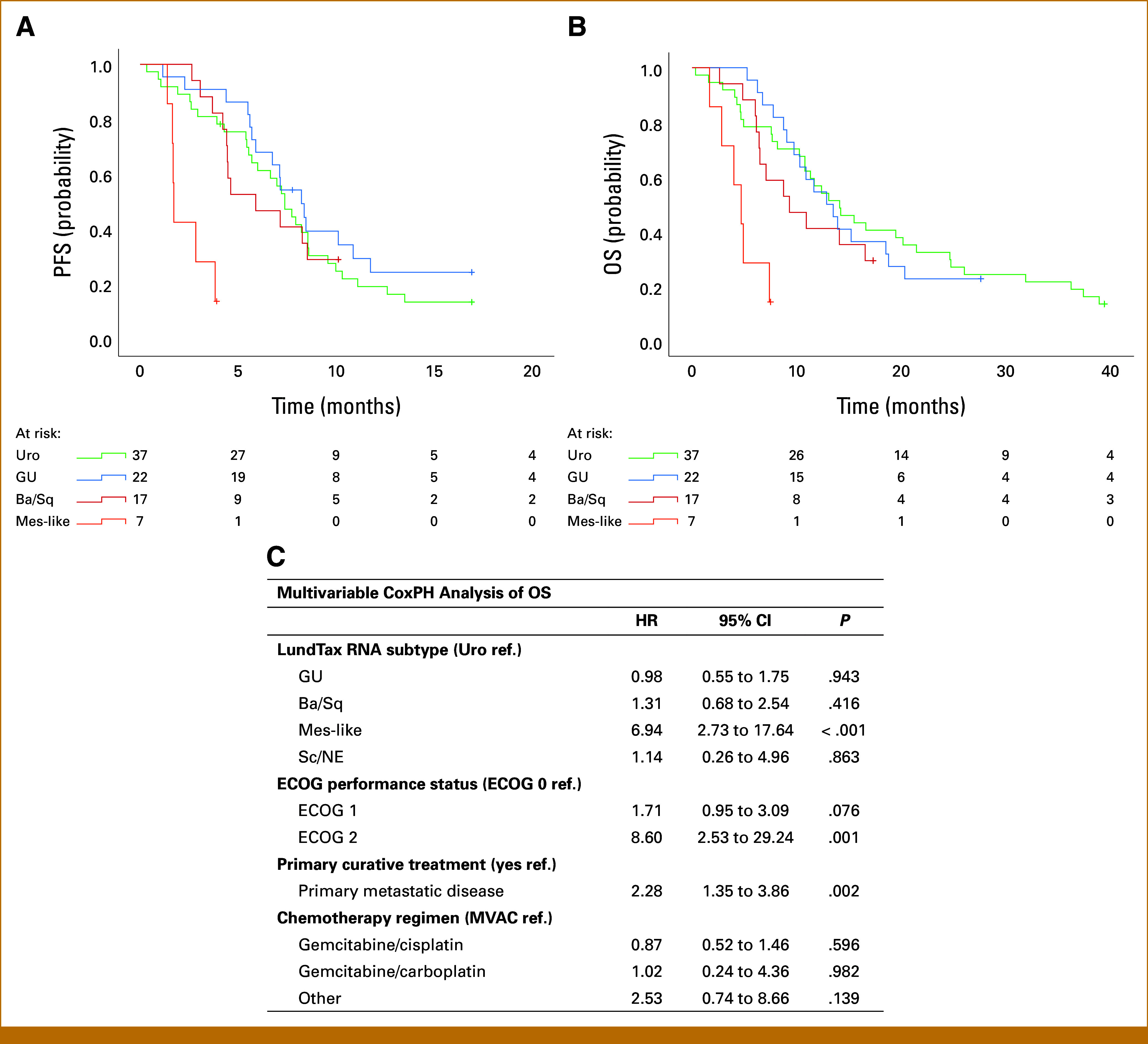
Survival by the RNA-based molecular subtypes. Kaplan-Meier curves for (A) PFS and (B) OS. The curves for PFS are separated with log-rank *P* < .001 and for OS with log-rank *P* = .004. The curves are truncated when the number at risk in any group falls below five. Sc/NE patients are excluded due to n = 3. (C) Multivariable CoxPH regression analyses showing shorter OS for Mes-like subtype independently of ECOG performance status, primary metastatic disease, and type of chemotherapy regimen. For survival analyses n = 86, nine patients had missing extracted RNA. Ba/Sq, basal squamous-like; CoxPH, Cox proportional hazards regression; ECOG, Eastern Cooperative Oncology Group; GU, genomically unstable; HR, hazard ratio; LundTax, Lund taxonomy; Mes-like, mesenchymal-like; MVAC, methotrexate, vinorelbine, doxorubicin, and cisplatin; OS, overall survival; PFS, progression-free survival; Sc/NE, small cell neuroendocrine-like; Uro, urothelial-like.

In the complete cohort, mPFS and median overall survival (mOS) were 7.2 months (95% CI, 6.0 to 8.4) and 12.4 months (95% CI, 10.0 to 14.8), respectively. Patients with the Mes-like subtype had shorter PFS compared with the other subtypes (non–Mes-like, ie, Uro, GU, Ba/Sq, and Sc/NE subtypes; HR, 5.18 [95% CI, 2.28 to 11.76], *P* < .001; Fig 2A). Similarly, OS was shorter for patients with the Mes-like subtype compared with the non–Mes-like subtypes (HR, 3.19 [95% CI, 1.45 to 7.03], *P* = .004; Fig 2B). For the Uro subtype, mOS was 14.1 months, correspondingly 12.9 months for GU, 11.0 months for Sc/NE, 9.3 months for Ba/Sq, and 4.7 months for Mes-like (Data Supplement, Table S7). When the Uro subtype was further subdivided, UroB showed shorter mOS of 10.8 months compared with Uro A (12.4 months) and Uro C (19.5 months). Furthermore, the Mes-like subtype was independently associated with shorter OS (HR, 6.94 [95% CI, 2.73 to 17.64], *P* < .001) adjusting for performance status, previous curative treatment, and type of chemotherapy regimen in multivariate CoxPH analysis (Fig 2C and Data Supplement, Table S8).

Applying LundTax IHC subtyping identified similar association between subtypes and OS (Data Supplement, Tables S7-S9). On the contrary, subtypes defined by the consensus classification showed no significant differences in OS (Data Supplement, Tables S7 and S8).

### Differential Gene Expression Analyses in Relation to Response and Survival

**FIG 3.**
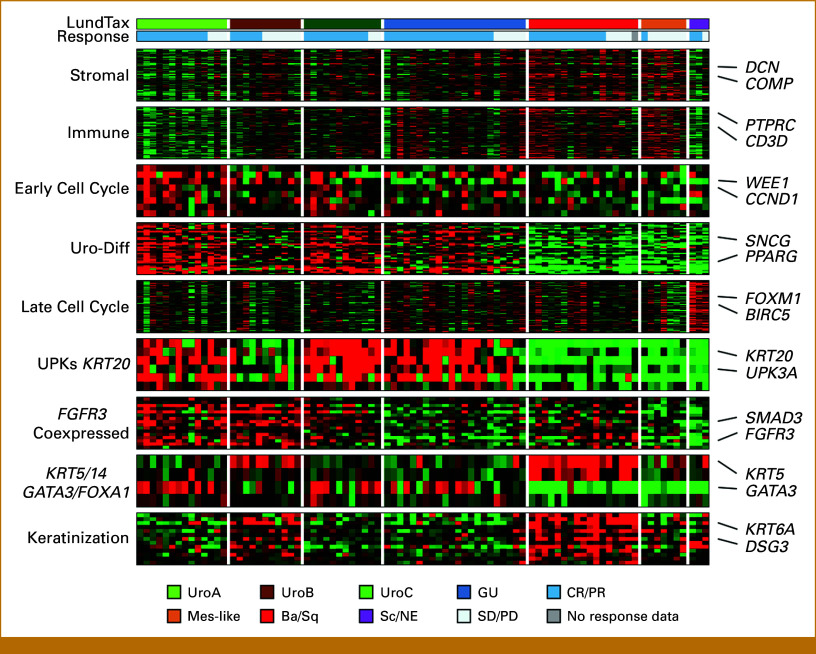
Transcriptomic analyses of response. Signatures associated with urothelial cancer biology show association with the LundTax RNA-based subtypes but not with response (CR and PR *v* SD and PD). The heatmap is median centered on 0 (black), and ranges from –0.9 (green) to +0.9 (red) relative log2 expression. Ba/Sq, basal squamous-like; CR, complete response; GU, genomically unstable; LundTax, Lund taxonomy; Mes-like, mesenchymal-like; PD, progressive disease; PR, partial response; Sc/NE, small cell neuroendocrine-like; SD, stable disease; Uro, urothelial-like.

For the 86 cases with transcriptomic data available, we ordered the cases by molecular subtype and response status (CR/PR *v* SD/PD) and visualized a set of subtype-specific gene signatures in a heatmap (Fig 3). This analysis revealed the expected associations between signatures and subtypes, for example, higher expression of urothelial differentiation (Uro-diff) genes among the luminal subtypes Uro and GU, higher expression of keratinization genes in the Ba/Sq subtype, and the highest expression of late cell cycle genes in the Sc/NE tumors. However, the analyses did not reveal any associations between these signatures and response within the molecular subtypes.

**FIG 4.**
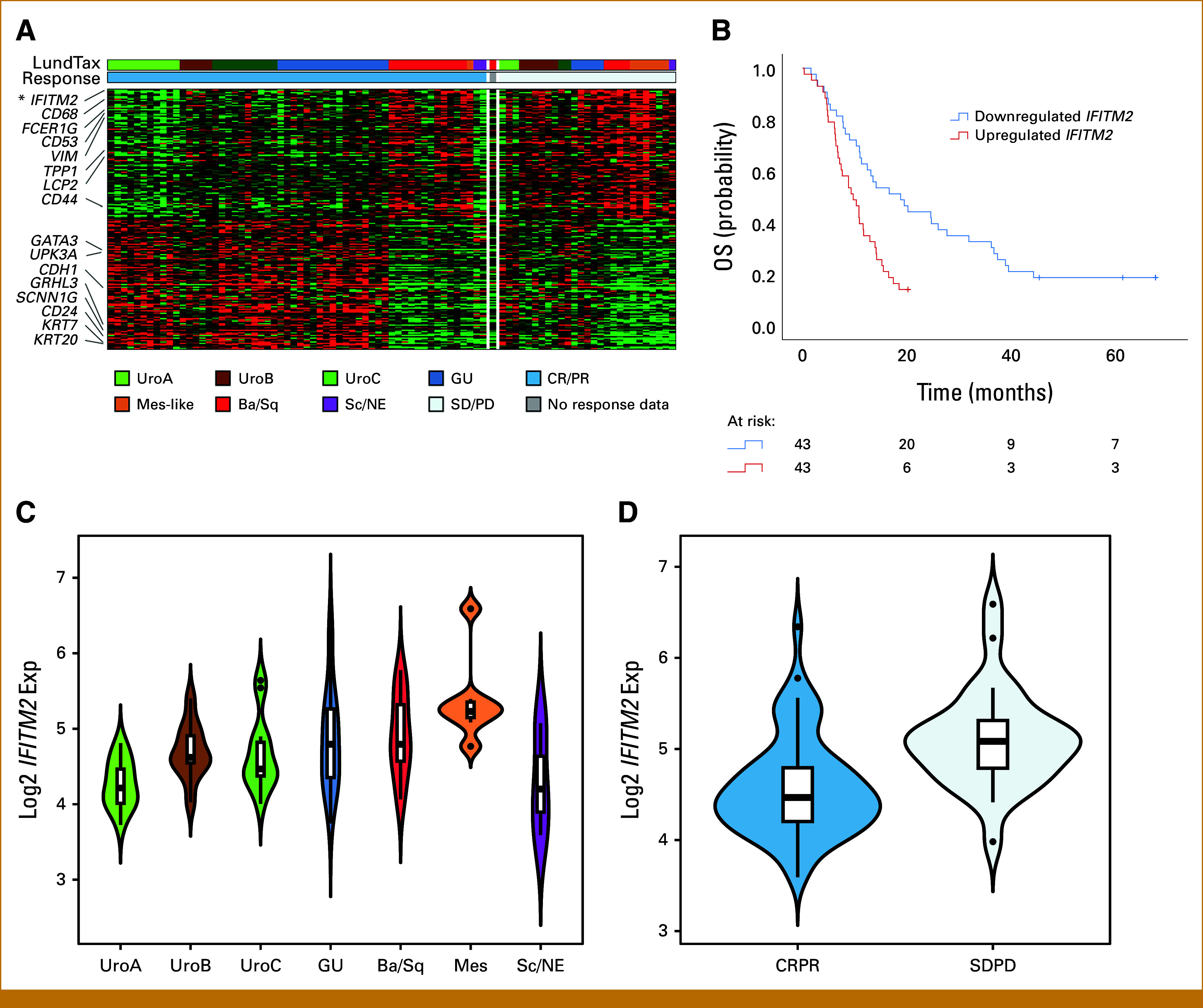
DEGs and signature expression analyses of response. (A) DEGs in the full cohort (n = 86) by response (CR and PR *v* SD and PD). *IFITM2* was the only significant DEG, with lower expression in responders. Genes in responders revealed an enrichment of top 100 downregulated stromal- and immune-related genes (eg, *IFITM2*, *CD68*, *FCER1G*, *CD53*, *VIM*, *TPP1*, *LCP2*, and *CD44*) and an enrichment of top 100 upregulated luminal urothelial and epithelial genes (eg, *GATA3*, *UPK3A*, *CDH1*, *GRHL3*, *SCNN1G*, *CD24*, *KRT7*, and *KRT20*). (B) Kaplan-Meier curves for OS show significant prolonged survival for patients with low expression of *IFITM2*, *P* = .007. The curves are truncated when the number at risk falls below five. (C) *IFITM2* expression in the different LundTax RNA molecular subtypes. (D) *IFITM2* expression in correlation to response (CR and PR *v* SD and PD). Ba/Sq, basal squamous-like; CR, complete response; DEGs, differentially expressed genes; GU, genomically unstable; LundTax, Lund taxonomy; Mes-like, mesenchymal-like; OS, overall survival; PD, progressive disease; PR, partial response; Sc/NE, small cell neuroendocrine-like; SD, stable disease; Uro, urothelial-like.

We then identified DEGs by response status in the full cohort, as well as in the luminal and nonluminal subtypes separately. Only one gene was significant, interferon-induced transmembrane protein 2 (*IFITM2*), with significantly lower expression in responders, where one unit increase in log2-IFITM2 expression was associated with an OR of 0.18, *P* < .001 (Figs 4A, 4C, 4D). Correspondingly, low expression of *IFITM2* was associated with improved mOS compared with high expression (18.8 months *v* 9.7 months, respectively, *P* = .007, Fig 4B). Additional analysis of the top 100 downregulated genes in responders revealed an enrichment of stromal- and immune-related genes (6 and 12 genes overlapped with the Stromal and Immune signatures, respectively, compared with the expected 0.7 and 0.8). Genes upregulated in responders were enriched for luminal and epithelial genes (Fig 4A), many of which are members of the Uro-diff signature or are known markers of (apical) urothelial cells. This increased luminal gene expression in responders was not completely explained by the lower response rates of the nonluminal subtypes, since the identical patterns of stroma/immune being linked to nonresponse and luminal genes being linked to response were also seen in the DEG analysis within the luminal subtypes (Data Supplement, Fig S3A). In the analysis within the luminal subtypes, only apolipoprotein D (*APOD*) with lower expression in responders reached significance. We also analyzed DEGs within luminal and nonluminal subsets. The analysis revealed that different DEGs were identified when the analysis was performed on these subsets, and that luminal DEGs were not associated with response in nonluminal cases and vice versa. From the DEG analysis within the nonluminal subtypes, the top 100 upregulated and downregulated genes seemed to identify the nonresponding Ba/Sq and Mes-like tumors (Data Supplement, Fig S3B). The DEGs identified in the full cohort showed a greater overlap with the luminal-specific DEGs than with the nonluminal DEGs (Data Supplement, Fig S3C). This suggests that molecular subtypes have largely different markers of response, and that, ideally, differential expression analysis of therapy response in UC should be performed separately for each subtype in addition to the full cohort. The complete list of DEGs is shown in the Data Supplement (Table S10).

Enrichment of gene ontology terms confirmed the association of stromal- and immune-related signatures to nonresponse (Data Supplement, Fig S4A and S4B). Eight genes were identified that were not linked to stromal or immune cell content, which may be candidate markers for resistance to palliative chemotherapy (Data Supplement, Fig S4C). We performed GSEA using the preranked algorithm to identify published signatures enriched among our detected DEGs. In total, 17 signatures were significantly enriched among genes positively associated with response (including several cell cycle and E2F-target signatures) and 208 signatures were significantly associated with nonresponse (including several epithelial-mesenchymal transition and invasion signatures). All response-enriched signatures and the top 20 non–response-enriched are shown in order of normalized enrichment score (NES; Data Supplement, Fig S4D).

## DISCUSSION

In this study, the molecular LundTax subtypes correlated with differential response and survival in patients with mUC treated with CHT. Patients with Mes-like tumors had lower response rate and shorter survival, while the Uro and GU subtypes displayed the highest proportion of response and longest survival. The survival benefits were significant for the LundTax molecular subtype classifications. Applying the consensus classification, a significantly lower response rate was observed in patients with Ba/Sq tumors, but subtypes were, however, not associated significantly to differences in survival.

The gene expression data revealed the expected gene signatures associated with urothelial cancer biology, confirming that the LundTax classification is valid also in a metastatic cohort, in addition to in the setting of non–muscle-invasive and muscle-invasive settings.^13^ Patients within the luminal subgroup (GU and Uro) showed the highest ORR and longest OS after palliative CHT. This observation is in line with our previous findings demonstrating that the GU and Uro subtypes were more responsive to neoadjuvant cisplatin-based chemotherapy in MIBC.^12^ Exploring potential differences in outcomes within the Uro group, we observe differences in both ORR and OS, which are suggested to be related to inherent molecular differences also within the Uro subtype. The UroB subtype appears to possibly represent a more aggressive subtype, a finding in line with previous observations in the neoadjuvant study. The Mes-like patients showed significantly lower ORR and shorter OS, independently of differences in baseline characteristics, suggesting the Mes-like subtype to be less responsive to palliative CHT and with worse overall prognosis.

Similarly, when applying the consensus classification, subtype-associated differences were observed in response rate, demonstrating significantly lower ORR for the Ba/Sq tumors. Furthermore, Ba/Sq tumors showed the shortest mOS among the consensus subtypes, although this finding was not significant. The consensus classification does not include a mesenchymal-like subtype but groups these together with Stroma-rich or Ba/Sq tumors (in this cohort, four Mes-like were Stroma-rich and three were Ba/Sq). Thus, the identification of poor responses in the consensus Ba/Sq subtype is partly due to the inclusion of Mes-like patients in this subtype category, all of whom were nonresponders. This highlights that details of various classification systems need to be taken into account applying molecular subtyping in translational studies.

We found that stromal and immune signatures were associated with response to CHT, in line with what have been suggested by others in previous studies.^6,28,29^ In our study, the nonluminal subgroup (Ba/Sq and Mes-like subtypes) was associated with higher expression of stromal- and immune-related gene signatures. Although *IFITM2* was the only gene significantly associated with response, the top 100 downregulated genes in responders revealed an enrichment of stromal- and immune-related genes indicating the importance of the tumor immune and extracellular matrix microenvironment in relation to CHT response in mUC. *IFITM2* is suggested to be involved in several functions in the immune and inflammatory systems.^30^ Furthermore, *IFITM2* has been reported to promote tumor progression and to be overexpressed in several types of cancer, such as in renal cell cancer where increased *IFITM2* expression was associated with unfavorable prognosis.^31^ In line with this, we found that downregulation of *IFITM2* was associated with favorable ORR and OS.

Although our study is based on a well-defined and population-based patient cohort, the sample size is still small in relation to the number of subtypes with consequently limited statistical precision in some of the analyses. Another study limitation was the use of primary tumor when assessing molecular subtypes that may deviate from subtypes found in the metastases.^32^ However, all patients were treatment-naïve at the time of commencing CHT, increasing the probability of the molecular subtype being the same between the initial sampling and the start of the treatment. Additionally, this study was done in a treatment-naïve mUC cohort and before systemic ICIs and ADCs were introduced. Unfortunately, to the best of our knowledge, there are no relevant cohorts available in the public domain that can be used to validate the present findings. However, this emphasizes the importance of our work since future studies in this space will be able to validate results in our publicly available data. If the main finding with a poor benefit from CHT in Mes-like tumors can be validated, the Mes-like subtype biomarker may be useful to deselect such patients for primary treatment with CHT in favor of other possible systemic treatment alternatives that are available today, including future ICI and ADC combinations to come.

In conclusion, LundTax molecular subtypes display differential clinical benefit from first-line CHT in a treatment-naïve cohort of patients with mUC. The Mes-like subtype was associated with both lower response and shorter survival, while the luminal subtypes displayed the best response rate and longest survival. Molecular subtypes hold promise as predictive biomarkers for developing precision medicine in mUC but need further validation in controlled prospective clinical trials with CHT, ICIs, and ADCs before clinical implementation.

## Data Availability

A data sharing statement provided by the authors is available with this article at DOI https://doi.org/10.1200/PO.24.00209. The data used in the current study are available from the Gene Expression Omnibus under the accession number GSE250194. Further information is available from the corresponding author upon request.
